# Trace element contamination in three shorebird species migrating through Delaware Bay, New Jersey: arsenic, mercury and selenium are increasing

**DOI:** 10.1007/s10646-024-02816-1

**Published:** 2024-10-29

**Authors:** Joanna Burger, Stephanie Feigin

**Affiliations:** 1 Division of Life Sciences, Environmental and Occupational Health Sciences Institute, Rutgers University, 604 Allison Road, Piscataway, NJ 08854 8082 USA; 2 Environmental Sciences Graduate Program, Rutgers University, New Brunswick, NJ USA; 3 Wildlife Restoration Partnerships, 109 Market Lane, Greenwich, NJ 08323 USA

**Keywords:** Trace elements, Shorebirds, Temporal patterns, As, Hg, Se

## Abstract

Many shorebird populations are declining, and contaminants may be partly contributing to the decline by interfering with feeding, migration, and breeding success. The goal of our study was to determine whether there was a temporal change in concentrations of trace elements of red knot (*Calidris canutus rufa*), sanderling (*Calidris alba*), and ruddy turnstone (*Arenaria interpres*) during spring migration in Delaware Bay, New Jersey, USA. We sampled blood to 1) determine levels of trace elements in 2019, 2) compare 2019 trace element levels with those from shorebirds in 2011–2012, and 3) examine variability in blood levels of trace elements among species. In 2019: 1) trace element levels were significantly different among species (except cadmium[Cd]), 2) Cd was lowest in all species, and arsenic (As) and selenium (Se) were highest, and 3) sanderlings had the highest levels of As and Se, and knots had the highest levels of chromium (Cr) and lead (Pb). Se was higher in these shorebirds than reported for other shorebirds from elsewhere. As, mercury (Hg), and Se increased significantly between 2011–2012 and 2019 in all three species. There were no significant temporal changes in Cd. Chromium (Cr) decreased in knots and sanderling. The temporal increases in As, Se, and Hg bear watching as they are toxic in vertebrates, and each can decrease the toxicity of the others. The data indicate that shorebirds can be bioindicators of changing trace element levels in estuaries, potentially providing early warning of increasing levels of As, Hg, and Se in the environment.

## Introduction

Identification of the threats at different stopover nodes on migratory pathways will allow managers and conservationists to determine which links present the greatest risk (Webster et al. 2002), especially for long distance migrants (Galbraith et al. 2014; Correll et al. 2016). Because many shorebird species are endangered or declining, it is important to identify the threats or stressors to their populations (Colwell 2010; Andres et al. 2012). Worldwide, many species of shorebirds are showing rapid declines (Morrison et al. 2001; Studds et al. 2017; Smith et al. 2023). Further, shorebirds make some of the longest migrations of any birds. For example, red knot (*Calidris canutus rufa*) can fly 15,000 km one way during migration (Minton et al. 2010; Minton et al. 2013; Niles et al. 2012; Smith et al. 2023). Understanding the threats shorebirds face at different nodes on the pathway between breeding and wintering sites is an important scientific and societal goal. Contaminants are one threat that requires additional study for migrating birds (Burger 1977; Goutte et al. 2014a, 2014b; Seewagen 2020; Ma et al. 2022). Reduced reproductive performance has been linked to As, Pb, and Hg in birds (Eisler 1991; Frederick and Jayasena 2010; Burger and Gochfeld 2010; Tartu et al. 2013; Adams et al. 2020; Ma et al. 2022). Reducing or controlling pollution requires laws and regulations, which require data on contaminant levels and effects in biota (Evers et al. 2011; Piersma et al. 2016). Understanding whether there are temporal changes in exposure, and whether there are species differences that reflect exposure, are both important to determining whether there are adverse health effects (Burger 2006; Egwumah et al. 2017; He et al. 2019).

In this paper, we determined the concentration of several trace elements (As, Pb, Hg, Cd, Cr, and Se) in three species of shorebird: red knot, sanderling (*Calidris alba*), and ruddy turnstone (*Arenaria interpres*). Red knots are endangered in Canada and federally threatened in the U.S. (USFWS 2014a, 2014b), and the other two species are declining rapidly (Andres et al. 2012). We used blood as the sampling endpoint to 1) determine levels of trace elements in 2019 (including correlations among trace elements), 2) compare trace element levels in 2019 with those from 2011–2012, and 3) examine the variability in blood levels among species. We were particularly interested in this time period because global atmospheric Hg emissions increased, which could be driven by factors such as increased coal-fired power plant production in China (Clark et al. 2023), and some decreased environmental regulations in the U. S. during the 2017-2020 period, allowing increased powerplant emissions (Mansfield 2021). However, it is difficult to draw direct comparison between levels in biota and those in air and water (Wang et al. 2019).

The three species mainly consume the same food source (horseshoe crab eggs, *Limulus polyphemus*) for the 2-3 weeks they are at Delaware Bay during their spring stopover on their way to Arctic breeding grounds, supplemented by other small invertebrates, such as *Donax* sp. (Tsipoura and Burger 1999). If they are using the same food source, their blood trace element concentrations should be similar, as blood levels reflect recent exposure (days to weeks) (Perkins et al. 2016; Burger et al. 2023). Samples were taken after birds were foraging in the bay. Blood levels of trace elements (except for As) in the blood of shorebirds are correlated with the levels in the eggs of horseshoe crabs at our sampling sites on Delaware Bay (Burger 2023). We tested the hypothesis that there are no temporal differences in trace element levels between the two time periods. Any increase in trace elements could indicate a potential health risk to shorebirds, other species consuming them, and to the food chain generally.

## Materials and methods

### Study site

**Fig. 1:**
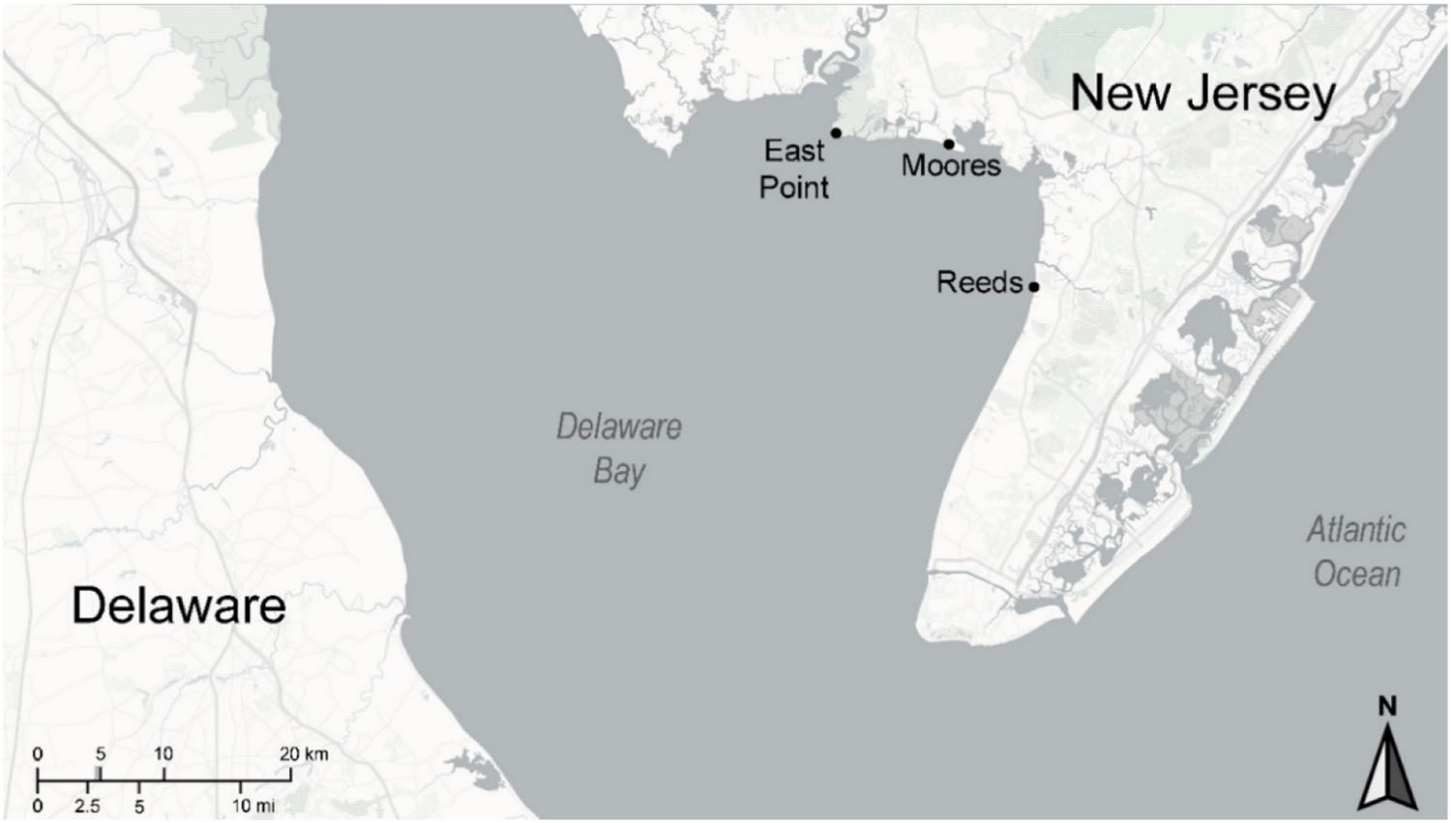
Map of Delaware Bay showing locations of blood collection in three species of shorebirds during migration in May 2019

Our protocol in 2019 was to collect blood from three species of shorebirds during their migratory stopover at Delaware Bay, New Jersey, USA in May (Fig. 1), for comparison with blood samples taken in 2011–2012 (Burger et al. 2017). Delaware Bay is one of the major ports along the U.S. Atlantic Coast and receives pollution from port and industrial activities, although the bay shows minimal human disturbance (Sutton et al. 1996). The trace elements in this paper were considered major elements of concern in the Sutton et al. review.

### Field data collection

Shorebirds were captured by cannon net and placed in holding cages protected from the sun (all held less than 2 h). About 100 microliters of whole blood was drawn from the cutaneous ulnar vein, using a 26 G ½ inch needle to puncture the vein. Blood was drawn into heparinized capillary tubes, and the capillary tubes were placed in non-additive vacutainers to prevent breakage (summarized from Burger et al. 2017). In keeping with humane practices, less than 1% of the body weight of blood was collected from each shorebird (Gaunt and Oring 1997). Blood was immediately frozen and brought back to the Environmental and Occupational Health Sciences Institute (EOHSI) of Rutgers University for analysis. Blood samples were collected from 20 – 32 birds per species in 2011–2012 (Burger et al. 2017) and 30–45 birds per species in 2019 (this study). All samples were collected under an approved protocol from the Rutgers University Institutional Animal Care and Use Committee (# 92-036, renewed every three years), and with appropriate state and federal permits. All birds were immediately released after blood collection.

### Chemical analysis

All samples were prepared and analyzed in laboratories of the EOHSI at Rutgers University. Separate laboratories were used for digestion and chemical analysis. All containers and equipment were washed and rinsed (10% HNO solution, deionized water) before any analysis. Blood from each bird was individually homogenized and digested in 70% TraceMetal™ grade nitric acid (Fisher Chemical) in a microwave (MDS 2000 CEM). Total Hg was analyzed using a Perkin Elmer FIMS-100 Hg analyzer. Other trace elements were analyzed with a Perkin Elmer 5100 Flameless Atomic Absorption Spectrometer (Zeeman correction). The instrument detection limits were 0.02 ng/g for As and Cd, 0.08 ng/g for Cr, 0.15 ng/g for Pb, and 0.02 ng/g for Hg and Se. To evaluate QA/QC, we used standard reference material (DORM-2) from the National Research Council Canada for Hg analysis, as well as spiked samples, blanks, and replicate samples. For the Zeeman graphite furnace Atomic Absorption Spectroscopy quality control we used DORM-2 Standard Reference material (SRM) 1460 (trace metals in water). Recovery rates ranged from 91 to 106%. The same methods were used in 2011/2012 and in 2019, by the same technician on the same instruments. Additional information on methods is published in Tsipoura et al. (2017), and in Burger et al. (2017 and 2019). Whole blood concentrations are expressed in ng/g (ppb, wet weight).

### Statistical analysis

In view of small sample sizes, Kruskal-Wallis X^2^ statistics were used to examine whether there were differences in trace element levels among species and among years, with Bonferroni correction (SAS 2020). We used Kendall Tau correlations to determine differences among shorebird species. Non-parametric tests were used because they are best suited for data sets with small sample sizes, and are more conservative (McDonald 2022).

## Results

Table 1 summarizes the blood concentrations of the six analytes for all three species of shorebirds. Overall, the element with the lowest level in blood of shorebirds was Cd and the highest was Se. The 2019 data on trace element levels demonstrates that: 1) there are species differences in all trace elements, except Cd, 2) red knots had the highest levels of Cr and Pb, 3) the levels of Cr and Pb in knots were an order of magnitude higher than the levels in the other species, 4) sanderlings had the highest levels of As, Hg, and Se, and 5) ruddy turnstones did not have the highest levels of any trace elements (Table 1).

**Table 1:** Comparison of trace element levels (ng/g, wet weight, ppb) among three species of shorebirds (2019) from Delaware Bay New Jersey

	Red knot	Sanderling	Ruddy Turnstone	X^2^ (p)
Sample size	30	34	45	
As	1688 ± 141 (1496 ± 145)	2304 ± 176 (2011 ± 205)	1813 ± 104 (1646 ± 122)	8.1 (0.02)
Cd	1.5 ± 0.4 (0.3 ± 0.1)	3.8 ± 1.4 (0.43 ± 0.2)	2.4 ± 0.5 (0.4 ± 0.2)	0.47 (NS)
Cr	224.6 ± 94.3 (111.8 ± 20.6)	36.2 ± 4.9 (21.6 ± 5.2)	38.6 ± 4.9 (28.1 ± 3.4)	34.5 (<0.0001)
Pb	235.1 ± 39.1 (131.5 ± 28.7)	66.7 ± 19.7 (22 ± 6.9)	50.5 ± 9.4 (15.1 ± 5.4)	24.9 (<0.0001)
Hg	92.1 ± 7.7 (82.1 ± 7.6)	203.8 ± 20.0 (182.4 ± 14.4)	116.9 ± 8.8 (103.6 ± 7.8)	35.1 (<0.0001)
Se	19,197 ± 2395 (15,007 ± 2072)	22,453 ± 2159 (18,610 ± 2143)	12,387 ± 1560 (8,438.2 ± 1194)	16.3 (0.0003)

Given are arithmetic means ± standard errors (geometric means below), and Kruskal-Wallis X^2^ tests

There were correlations between trace elements for the three species of shorebirds. There were 15 possible correlations per species (=45 possibilities), and there were only 8 positive correlations, and most were not strong (Table 2). No correlation was significant in all three species. Hg is the only trace element that was not correlated with any other trace element.

**Table 2:** Kendall Tau correlations among trace elements for three species of shorebirds migrating through Delaware Bay in 2019

		As	Cd	Cr	Pb	Hg	Se
Red Knots (N = 29)	As	–					
	Cd	0.25	–				
	Cr	0.18	−0.04	–			
	Pb	0.56**	−0.04	0.0003	–		
	Hg	−0.12	0.23	−0.15	−0.12	–	
	Se	0.33	0.31	0.18	0.37*	−0.35	–
Sanderling (N = 32)	As	–					
	Cd	−0.18	–				
	Cr	0.08	0.48**	–			
	Pb	0.07	0.75**	0.67**	–		
	Hg	−0.13	−0.16	0.01	−0.13	–	
	Se	0.46**	−0.23	−0.11	−0.13	−0.06	–
Ruddy Turnstone (N = 43)	As	–					
	Cd	0.28	–				
	Cr	0.26	0.04	–			
	Pb	0.13	0.33*	0.26	–		
	Hg	−0.23	−0.05	−0.03	−0.11	–	
	Se	0.31*	0.19	0.27	0.15	0.04	–

*p < 0.05; **p < 0.01

### Temporal differences

For knots, levels of As, Pb, Hg, and Se increased significantly from 2011-2012 to 2019, and Cr decreased (Table 3). For sanderlings, levels of As, Hg, and Se increased significantly, while Cr declined. For ruddy turnstones, Hg and Se increased significantly. Of particular interest was that As, Hg and Se increased significantly from 2011/2012 to 2019 (almost 3-fold) in all three species. Given are arithmetic means ± standard errors (and geometric means ± standard errors) and range. GLM with Bonferroni Correction.

**Table 3:** Temporal comparison of trace element levels (ng/g, wet weight) (ppb) in shorebirds from 2011–2012 and 2019 from Delaware Bay, New Jersey

Species	Trace element	2011–2012	2019	X^2^ (p)
Red knot	Sample size	30	30	
	Mean weight of bird	147.9 ± 6.1 (144 ± 6.2); 98–204	152.8 ± 8.1 (154.3 ± 6.4); 105–202	1.33 (NS)
	As	866 ± 82 (806 ± 95); 270–1800	1688 ± 141 (1496 ± 145); 370–3400	7.6 (0.008)
	Cd	2.3 ± 0.3 (1.4 ± 0.4); 0–6.7	1.5 ± 0.4 (0.3 ± 0.1); 0.01–7.7	0.18 (NS)
	Cr	488 ± 63.4 (384.7 ± 48.6); 110–1700	224.6 ± 94.3 (111.8 ± 20.6); 21–2920	5.4 (0.02)
	Pb	88.9 ± 12.6 (58.7 ± 15); 0.1–310	235.1 ± 39.1 (131.5 ± 28.7); 21–620	12.9 (0.0007)
	Hg	16.5 ± 3.1 (8.2 ± 2.0); 0.3–64.1	92.1 ± 7.7 (82.1 ± 7.6); 26.3–178	85.4 ( < 0.0001)
	Se	5912 ± 591 (4859 ± 605); 870–13,000	19,197 ± 2395 (15,007 ± 2072); 2200–57,000	30.2 ( < 0.0001)
Sanderling	Sample size	20	34	
	Mean weight of bird	73.3 ± 3.2 (72 ± 3.2); 52–98	65.4 ± 4.4 (66.7 ± 3.5); 41–98	0.52 (NS)
	As	1288 ± 193.4 (1053 ± 152); 390–3500	2304 ± 176.2 (2011 ± 205); 340–3400	13.8 (0.0005)
	Cd	2 ± 0.7 (0.4 ± 0.2); 0–11	3.8 ± 1.4 (0.43 ± 0.2); 0.01–40	0.9 (NS)
	Cr	122.2 ± 20.9 (96.4 ± 14.9); 29–370	36.2 ± 4.9 (21.6 ± 5.2); 0.1–110	25.1 ( < 0.0001)
	Pb	87.1 ± 13.7 (65 ± 12.1); 14–185	66.7 ± 19.7 (22 ± 6.9); 0.1–460	0.5 (NS)
	Hg	24.8 ± 5.3 (12.6 ± 4.0); 1–80	203.8 ± 20.0 (182.4 ± 14.4); 56.5–743	45.5 ( < 0.0001)
	Se	14,500 ± 2306 (11,704 ± 2011); 3000–35,000	22,453 ± 2159 (18,610 ± 2143); 3900–52,000	5.3 (0.03)
Ruddy turnstone	Sample size	32	45	
	Mean weight of bird	121.3 ± 3.2 (120 ± 3.1); 87–167	118.9 ± 6.2 (120.1 ± 5); 77–198	0.2 (NS)
	As	506.2 ± 34 (471 ± 32.3); 190–1000	1813 ± 104 (1,646.5 ± 122); 220–8600	45.2 ( < 0.0001)
	Cd	5 ± 0.8 (2.6 ± 0.8); 0–21	2.4 ± 0.5 (0.4 ± 0.2); 0.01–140	0.2 (NS)
	Cr	272 ± 40.1 (184 ± 31.8); 17–990	38.6 ± 4.9 (28.1 ± 3.4); 5.7–11,000	0.0 (NS)
	Pb	50.5 ± 9.4 (109.5 ± 15.4); 32–650	50.5 ± 9.4 (15.1 ± 5.4); 0.1–340	16.8 (0.0001)
	Hg	39.9 ± 6.7 (13.1 ± 5.4); 0.1–130	116.9 ± 8.8 (103.6 ± 7.8); 27.6–279	46.3 ( < 0.0001)
	Se	6294 ± 786 (4684 ± 709); 790–18,000	12,387 ± 1560 (8438 ± 1194) 1100–47,000	9.4 (0.003)

**Fig. 2:**
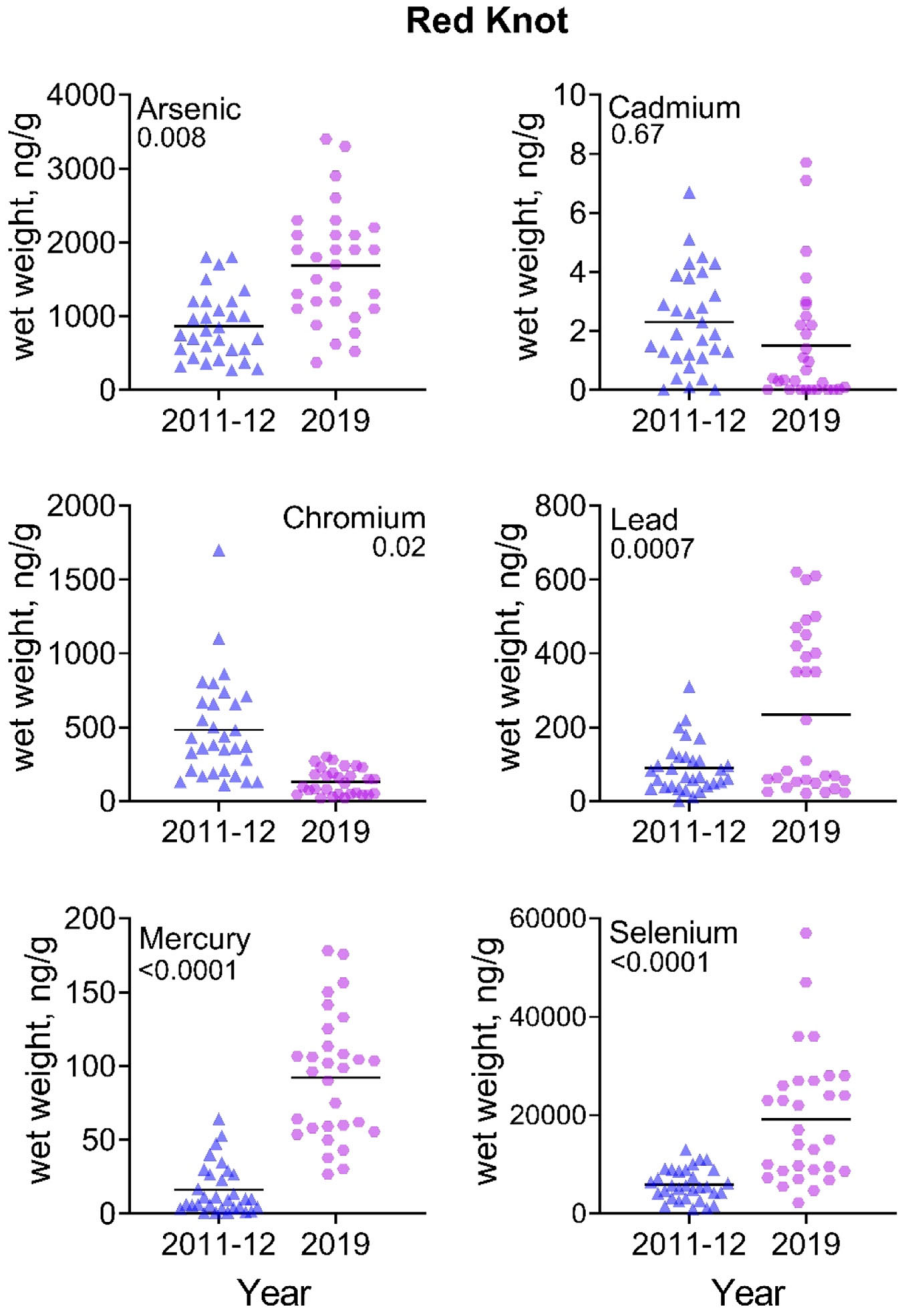
Comparison of blood trace element concentrations (wet weight, ng/g) in red knots migrating through Delaware Bay in May 2011/2012 and 2019. The line through each dataset is the median for that category

**Fig. 3:**
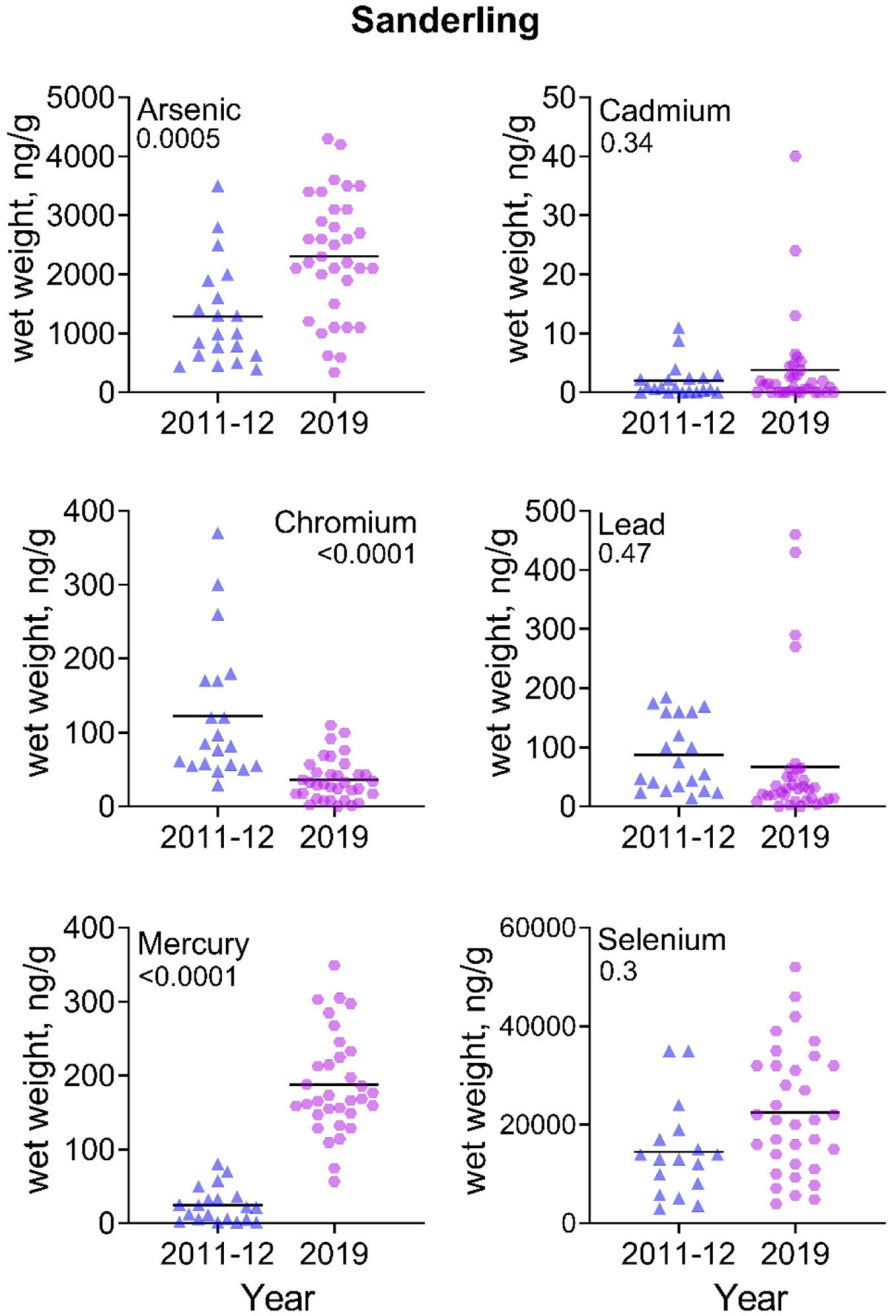
Comparison of blood trace element concentrations (wet weight, ng/g) in sanderlings migrating through Delaware Bay in May 2011/2012 and 2019. The line through each dataset is the median for that category

**Fig. 4:**
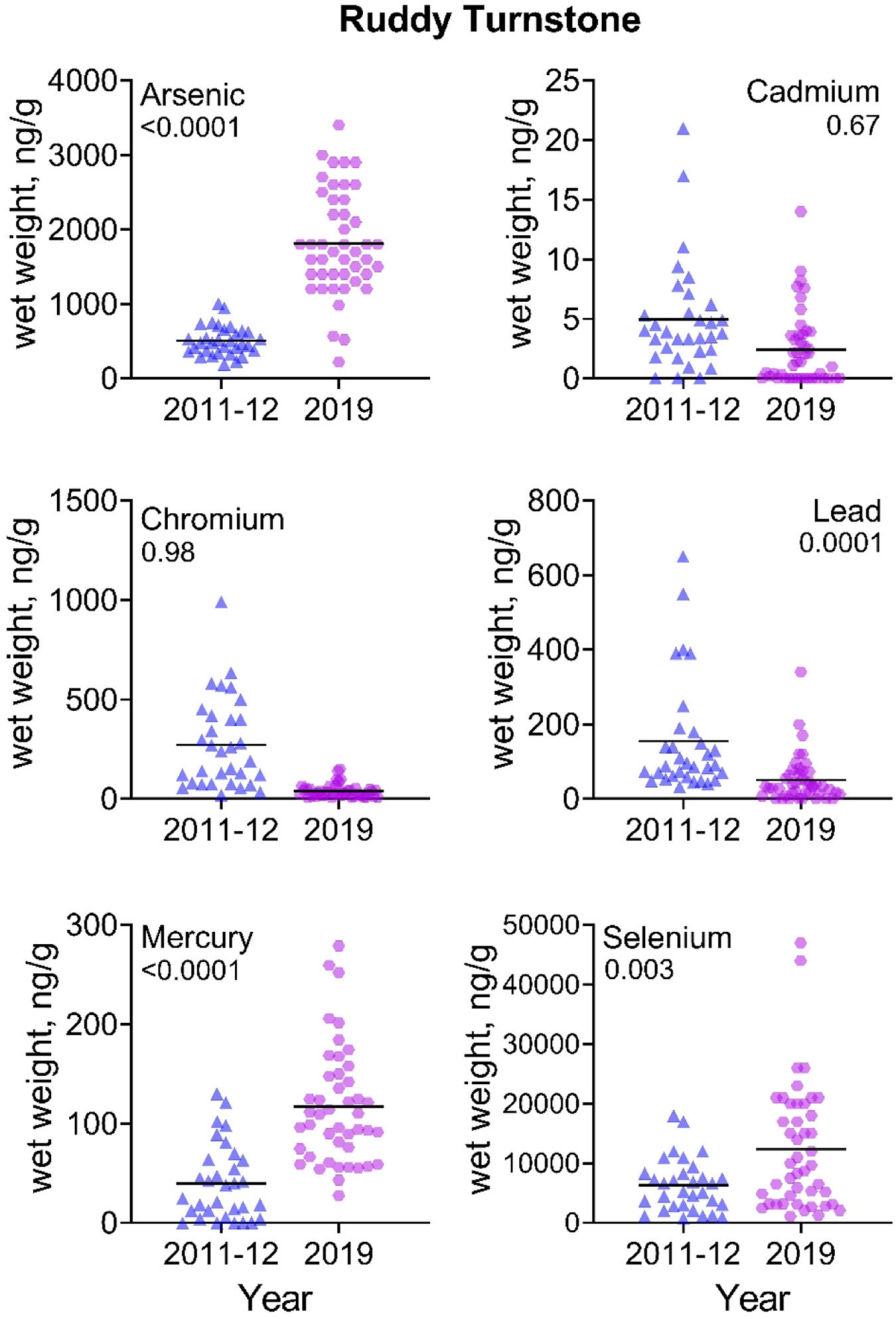
Comparison of blood trace element concentrations (wet weight, ng/g) in ruddy turnstones migrating through Delaware Bay in May 2011/2012 and 2019. The line through each dataset is the median for that category

A visualization of the data provides a clearer picture of the range of trace element levels in each species for 2011/2012 compared to 2019 (Figs. 2–4). For some trace elements, there is little variation and the trace element concentrations for all individuals are rather similar. For example, the concentrations of Cr in turnstones and knots in 2019, Pb in 2011/2012 in knots, Hg in knots and turnstones in 2011/2012, and Se in knots in 2011/2012, among others, exhibited great variability in concentrations. For other trace elements, there is great variation in levels within a species (for example, As in all three species in 2019). For some, there are several outliers in several trace elements (e.g., Cd, Pb, and Se in knots, thus the reason for presenting geometric means in the tables).

## Discussion

The main goal of this research was to determine if the concentrations of trace elements had changed since the 2011-2012 sampling period when contaminant concentrations were last examined in these shorebirds. The most significant finding in this report was the increase of some trace element concentrations in the blood of shorebirds from 2011/2012 to 2019, especially for As, Se and Hg. Secondly, there were differences in all trace element concentrations (except Cd) in the blood among the three species of shorebirds during their critical migratory stopover at Delaware Bay while on their way to northern breeding grounds. There were no positive correlations among any two metals that occurred for all three species, although for Sandering and Turnstones, lead and cadmium, and selenium and arsenic were correlated. It is also useful to compare the levels found in Delaware Bay with those for shorebirds from elsewhere to provide context for the significance of levels. Each of these topics will be discussed below.

### Temporal patterns

As, Hg, and Se increased in all three species of shorebirds migrating through Delaware Bay from 2012 to 2019. Hg increased by an order of magnitude in sanderlings and tripled in ruddy turnstones, and As and Se doubled or almost doubled in all three species. These are large increases for trace elements over less than a decade and suggest the importance of continued sampling at appropriate intervals to determine the severity of this change. Possible reasons for the temporal increases in Hg and Se in the blood of shorebirds could be: 1) increases in agricultural runoff or atmospheric deposition of Hg to the waters of the bay (NADP 2023); if mercury increases without increases in Se (that partly mitigates the adverse effects of Hg) possible adverse effects of Hg might not be reduced, 2) changes in levels in the water (and subsequently horseshoe crab eggs) because of increases in upstream pollution (e.g. local pollution, Sutton et al. 1996; Ackerman et al. 1998; DRBC 2023), and 3) changes in avian foraging behavior or absorption into the blood stream that resulted in these differences. While not often investigated in laboratory experiments, trace elements can have an adverse effect on shorebirds (Ma et al. 2022). Hargreaves et al. (2011) did note that Se and Hg are bioconcentrated in shorebird blood compared with levels in local invertebrates.

Atmospheric deposition may have increased between 2012 and 2019 because of changes in U.S. Hg regulations. During the years from 2017-2020 there was a decrease in new regulations and a lessening of existing environmental regulations in the U.S. (Mansfield 2021). Much of the Hg deposition in the eastern U.S., for example, comes from coal-fired power plants in the Midwest (Morin and Miller 2022; NADP 2023). Pb and Cd have generally decreased in the environment over the last few decades, although trends in Hg have varied (ATSDR 1999, 2007, 2012, 2013). In contrast, Streets et al. (2019) reported an increase in global emissions and concentrations of Hg in the period 2010 to 2015. Sunderland et al. (2009) reported increases in Hg in the Northern Pacific Ocean, and more such studies are essential in the Northern Atlantic Ocean to understand local patterns or changes in Hg globally, particularly in the last decade. Partly, the question is one of time – atmospheric deposition and land-based pollution enter waters, into the sediment, and eventually into biota at the base of the food chain (Burger and Gochfeld 2016). One question we examined is whether there was a recent increase in any of the trace elements in Delaware Bay that we measured? The increase in Hg and Se may be an indication of increases in the Bay generally, or in the region generally. Se increased in horseshoe crab eggs in 2019 as well (Burger 2023).

Delaware Bay is one of the major shorebird migratory stopover sites (Niles et al. 2008). Habitat loss, predators, human disturbance, and contaminants could all play a role in foraging success at important stopover sites (Galbraith et al. 2002, 2014; Goss-Custard et al. 2006; Burger and Niles 2013, 2017). Any changes in habitat availability, predators, or contaminants could also impact survival and reproduction. Knots and other birds that reach the Arctic with low weights do not breed successfully (Baker et al. 2013). The loss of mudflats predicted in many bays because of sea level rise (Galbraith et al. 2002, 2014), including Delaware Bay, will have significant effects on the foraging success of shorebirds because they will have less mudflat space for foraging. Human disturbance can also have a significant effect on their use of habitats that are otherwise suitable (Burger and Niles 2013, 2017). Predators, such as peregrine falcon (*Falco peregrinus*), have had a significant effect on the behavior and migration of shorebirds (Lank et al. 2003; Dekker et al. 2011; Watts and Truitt 2021). Similarly, changes in contaminant levels could have sublethal effects.

To reach the Arctic in a healthy condition, knots and other shorebirds almost double their weight before leaving New Jersey (Baker et al. 2004, 2013; Morrison and Hobson 2004; Morrison et al. 2007; McGowan et al. 2011). While in Delaware Bay, they eat horseshoe crab eggs almost exclusively (Tsipoura and Burger 1999; Niles et al. 2008, 2009). There is a clear relationship between weight gain in Delaware Bay and success on the breeding grounds (Morrison and Hobson 2004; Duijns et al. 2017; Burger et al. 2022). These relationships allow for an understanding of how shorebirds successfully engage in a connectivity web of different stopover sites between their wintering and breeding sites, to allow successful breeding. While the effects of prey abundance and availability on breeding success have been well established, the relative effects of trace elements have not, but see Goutte et al.(2014a,b). Tracking changes in trace element levels is important to understanding possible deleterious effects.

The data indicate that blood of shorebirds may be a useful indicator of changing levels of contaminants because the response is rapid (levels in blood reflect levels in horseshoe crab eggs, their primary prey in the Bay, Burger et al. 2017) and the blood of shorebirds can be monitored regularly as they consistently migrate through the bay in May. Additionally, horseshoe crab eggs are eaten by young fish (Sutton et al. 1996; Burger et al. 2018), which are eaten by even larger fish that are eaten by people. Therefore, trace elements in crab eggs and shorebirds can act as sentinels of exposure for humans as well, particularly for mercury that bioaccumulates.

### Differences in trace element levels among species in 2019

Trace elements enter Delaware Bay through runoff from industrial and chemical processes, mining, and agriculture, as well as energy generation and atmospheric deposition (Furness and Rainbow 1990; Fitzgerald et al. 2005; Hoffman et al. 2011). Birds are then exposed through the food and water they consume, as well as through external exposure. Horseshoe crab eggs often are in water as the shorebirds consume them, providing additional exposure.

We expected that trace element concentrations in the blood of the three shorebirds would be similar in 2019 because they were eating predominantly the eggs of horseshoe crabs (Tsipoura and Burger 1999), and there is a positive relationship between the levels of trace elements in horseshoe crab eggs and levels in the blood of shorebirds (Burger et al. 2017, 2023). We suspected that turnstones might have different concentrations because they can dig down into the sand to reach intact crab egg clutches. That is, clutches may have different levels of trace elements because the females deposit an exudate when they lay their eggs that binds the eggs together in a clutch (along with some sand grains) (Smith et al. 2023). This exudate may contain additional metals, but this was not the case.

Eggs become available to shorebirds on the sand surface: 1) when the eggs are forced to the surface by other female crabs that dig up nests of females that had laid earlier, 2) the surf exposes some nests laid too near the low tide line, and 3) actions of the surf on nests higher on the beach (e.g. bioturbation: Smith 2007; Nordstrom et al. 2006; Smith et al. 2022). Only ruddy turnstones can dig down to reach clutches below the surface (in other words, clutches with exudate), although other species try to parasitize the turnstones. We found, however, that turnstones did not have the highest levels of any trace elements, and they had the lowest levels of Pb and Se (Table 1). In fact, red knots had the highest levels of Cr and Pb, and sanderlings had the highest levels of As, Hg, and Se. When sanderling and knots don’t have horseshoe crab eggs to gorge on, which happens very infrequently, they sometimes migrate to beaches north of the sampling area; elsewhere they eat a variety of marine invertebrates, and may vary their diet during their New Jersey sojourn as well (e.g., Cape Cod, L. Niles, pers. comm.). The levels of Cr in knots were an order of magnitude higher than that of the other species, but there were no other order of magnitude differences for the other trace elements. The greater levels of Cr in knots may occur because they feed in different parts of the Bay, or they were foraging for longer (e.g., number of days) on horseshoe crab eggs and were thus exposed more to these trace elements. The differences in the other trace elements, however, although significant, may not have been great enough to result in a meaningful biological difference.

As and Se were uniformly high in all three species. Dissolved arsenate predominates in open water as well as right along the shore (Yuan et al. 2021) that is frequented by horseshoe crabs and shorebirds. One explanation for the high levels of As in shorebird blood in 2019 may be the incorporation of particulate As in horseshoe crab eggs when they are at the surface. A similar mechanism might occur for Se. In Delaware Bay and its associated marshes, Se is largely from agricultural activities and runoff, as well as from atmospheric deposition (Velinsky and Cutter 1991; Sutton et al. 1996).

### Geographic comparisons among shorebirds

Examining trace elements in the blood of shorebirds is uncommon – feathers are an easier tissue to collect and process. However, there are some studies with whole blood, and the values from these studies are given in Table 4. We have also examined levels of trace elements in blood of semipalmated sandpipers (*Calidris pusilla*) in Delaware Bay (2012) and in Surinam, SA (2013) (Burger et al. 2018). Levels of all trace elements from Surinam in 2013 were lower than we found in the present study for the other three species of shorebirds (except for Cd) migrating through Delaware Bay in 2019 (Table 4). We had expected Hg levels in blood to be higher in Surinam because they have widespread gold mining and extensive agriculture (Burger et al. 2018). In contrast, the levels of trace elements in blood of semipalmated sandpipers from Brazil (2016) were higher than in Surinam, but Se was higher, and Hg and As were lower than we found in 2019 for the three shorebird species we studied in Delaware Bay. The studies in New Jersey, Surinam, and Brazil were conducted using the same methods on the same instruments.

**Table 4:** Some comparative levels (ng/g, wet weight) for blood of shorebirds from elsewhere, that migrate through Delaware

	Sampling Year	Sample Size	As	Cd	Pb	Hg	Se
Hargreaves et al. (2010/2011)							
Dunlin (Nunavut, Canada)	2009	5	18	NA	18	193	575
Ruddy Turnstone (Nunavut, Canada)	2009	36	10	NA	10–19	587	1221
Perkins et al. (2016)							
Dunlin (Alaska)	2008–2009	94	NA	NA	NA	200	NA
Semipalmated Sandpiper (Alaska)	2008	35	NA	NA	NA	750	NA
Burger et al. (2018)							
Semipalmated Sandpiper							
Surinam	2013	71	212	4	109	18	5330
Brazil	2016	62	455	2	97	20	29,700
New Jersey	2011–2013	30	381	2	60	13	4360
Burger and Feigin, this paper							
Red knot (Delaware Bay)	2019	30	1688	2	235	92	19,197
Ruddy Turnstone (Delaware Bay)	2019	34	1813	2	51	117	12,387
Sanderling (Delaware Bay)	2019	45	2304	4	67	204	22,453

Shown are means in ppb

*ND* non detect, *NA* not available

In comparing the levels of several trace elements with those from other studies (Table 4), we found that the levels from Nunavut were lower for As, Pb, and Se, but higher for Hg. Mean concentrations of Hg in blood ranged from 200 ppb to about 750 ppb in Alaska (Perkins et al. 2016). Thus the Hg levels in the present study were similar to, or lower than others reported.

The high levels of Se in the blood of the three species of shorebirds in Delaware Bay and in Brazil (Burger et al. 2018) are of interest as Se is highly regulated in the body around a very narrow range, at least in mammals (ATSDR 1996). Se is often considered beneficial, particularly for a presumptive benefit against Hg toxicity, but Se is toxic in its own right (ATSDR 1996; Heinz 1996). While the high Se levels could be due to differences in prey consumed, Se increased markedly in the blood of shorebirds in Delaware Bay in 2019, even though in both years they were eating the same prey items (horseshoe crab eggs). In contrast, Eurasian oystercatchers (*Haematopus ostralegus*) had red blood cell levels of about 15 ppm (=15,000 ppb) collected from the Waddenzee (Goede 1993). Hoffman (2002) reported toxic Se effects at levels in several aquatic birds of 28 ppm (=28,000 ppb); in our study sanderling averaged 22,000 ppb – certainly cause for concern. Se contamination is a global problem (Werkneh et al. 2023). The relative lack of studies on levels of trace elements in blood of shorebirds indicates a need for further studies that examine levels both temporally and spatially (Pratte et al. 2020).

## Data Availability

No datasets were generated or analysed during the current study.
